# A Case of Opium Poisoning

**Published:** 1876-05

**Authors:** A. R. Alley

**Affiliations:** Atlanta, Ga.


### A CASE OF OPIUM POISONING.

By A. R. ALLEY, M.D., Atlanta, Ga.

A was called in consultation with Dr. W. R. D. Thompson, of this city, to see a young lady, Miss ——, aged 18 years, who had taken one and a half ounces of laudanum. Dr. Thompson arrived some twenty-five minutes after she had taken the above named quantity, and found her almost in a moribund condition, with symptoms of an alarming nature, and in a semi-comatose state; pulse about 30 beats per minute; contracted pupils (small pin-hole), with excessive congestion of conjunctiva; stertorous breathing, with extremities cold and livid; with cutes anserina. We gave her sulph. zinc, grains xx, pulv. ipecac., grains xv, until free emesis was established. Being impossible to procure a stomach pump, we resorted to this method, and facilitated free emesis by giving her warm water until we were confident that the stomach was most thoroughly evacuated. We then gave her a solution composed of—

℞.—Sulph. atropia, . . . . . grs. 10-17

Aqua, dist., . . . . . . ℥ 40-17

Sig.—Teaspoonful.

Gave her a teaspoonful at a dose every fifteen minutes until 1½ grains had been taken by the mouth; the pupils of eyes dilated; increased pulse; using mustard cataplasms to wrists and thighs, along with flagellations with wet towels, constantly kept up until the reaction had commenced; pulse increased and skin became warm. Whisky was then given her, in free and large doses, until reaction was thoroughly restored and all toxical effects of the opium subsided.

This young lady was never in the habit of taking any preparation of opium. Being a "love affair, she had resorted to this method of committing suicide.

This case presents one of the most aggravated of opium poisoning. The large quantity taken, and some time having elapsed before any medical aid was summoned, would this not be considered a case of extreme poisoning? Yet it was controlled and its toxical effects arrested by prompt and large doses of atropia. The symptoms from the commencement were of the gravest character, and called for a quick and heroic remedy, which was administered, and a speedy recovery resulted, and the life of a young person was saved by a prompt and certain antidote for opium.

