# Correction

**Published:** 1876-06

**Authors:** A. R. Alley

**Affiliations:** Atlanta, Ga.


*Editors Atlanta Medical and Surgical Journal:*

In the last issue of your Journal for the month of May, a report of a case of opium poisoning was written by me, in which their appeared a grave error in the prescription, which read thus: ℞.—Sulph. atropia, grs. 10–17; aqua dist., ℥ 40–17, which should have been: ℞.—Sulph. atropia, grs. ij.; aqua dist., ℥ij. Sig. Teaspoonful; which would have been correct, as written in my original MSS. You will oblige me by correcting this great error in your next month's Journal.

Respectfully yours, A. R. Alley, M.D.

*Atlanta, Ga., May* 12, 1876.

